# Does the Translation Continuation Task Exhibit Interaction and Alignment Effects? Evidence from a CSL Classroom in Cambodia

**DOI:** 10.3390/bs16030351

**Published:** 2026-03-02

**Authors:** Huan Zhang

**Affiliations:** 1 College of Humanities and Law, South China Agricultural University, Guangzhou 510642, China; huan0914@scut.edu.cn; 2 College of Chinese Language and Culture, Jinan University, Guangzhou 510610, China

**Keywords:** Continuation Argument, translation continuation task, interactive alignment, promoting learning effects

## Abstract

The Continuation Argument, a newly emerging perspective on language acquisition, requires further exploration to deepen our understanding of how continuation-based tasks facilitate foreign language learning. This study examines the use of observable language forms within the integrated pedagogical procedure of the translation continuation task in Chinese as a second language (CSL) learning. Data were collected from 60 learners attending Khmer-Chinese translation classes in Grade 8 at a Chinese school in Phnom Penh, Cambodia. The findings reveal a consistent pattern of language reuse. (i) Learners demonstrate a significant increase in their reuse of target Chinese language structures (e.g., words, grammar, and discourse knowledge) from the pre-reading materials when completing the translation continuation tasks. (ii) The translation continuation task helps reduce errors and improve the quality of Chinese translations. (iii) Both teachers and students generally recognize the positive impact and pedagogical value of the translation continuation task. The observed “language reuse” is discussed in light of multiple potential mechanisms, such as priming and pedagogically induced imitation. Thus, the translation continuation task proves to be an effective method for guiding learners’ attention to and reuse of target language forms in practical CSL translation teaching.

## 1. Introduction

Under the impetus of globalization and informatization, and coupled with rapid economic development in contemporary China, language communication between China and other countries has increased remarkably. An active market for CSL translation has emerged, characterized by an evident increase in demand for CSL translation services. Consequently, there is an urgent need for a number of highly skilled translators specializing in Chinese who possess better translation abilities and adaptability to meet diverse purposes. However, CSL translation teaching currently faces several challenges, including a lack of systematic instruction, inappropriate teaching methods, insufficient emphasis on pre-translation reading, and unsatisfactory teaching outcomes. To effectively address these issues, scholars are actively exploring innovative teaching concepts and methods to enhance the efficiency and quality of CSL translation instruction, aligning with the evolving demands of the international Chinese translation profession.

The concepts of interaction and alignment have received increasing academic attention in recent years, leading to the application of related methodologies in language learning and teaching. Alignment was initially proposed by Pickering and Garrod (2004) as a mechanism that facilitates smooth interpersonal communication and arises from interaction. If interaction is weak, alignment becomes weak; conversely, if interaction is strong, alignment tends to be strong (C. Wang, 2010). Atkinson et al. (2007) emphasized that alignment occurred not only among individuals but also between individuals and interactive variables, such as physical environment, social context, and tools. C. Wang and Wang (2015) further revealed that interaction and alignment were significant at the level of linguistic structures, which could be induced by imitating language expressions. The aforementioned research suggests a causal relationship between interaction and alignment, offering a new perspective for understanding CSL translation teaching and learning.

The Continuation Argument is a novel perspective on language acquisition that posits language can be acquired through “continuation” and emphasizes achieving linguistic alignment via interactive language use (C. Wang, 2016a, 2021). C. Wang (2016a) proposed the translation continuation task, which can be regarded as a new pedagogical innovation that integrates the concept of “continuation” into translation teaching. Xu (2016) first applied this approach in English as a second language (ESL) translation teaching and conducted empirical research to examine its promotion of language learning. Although some relevant studies have been conducted, research on the role of continuation in CSL translation teaching remains limited. Given that ESL and CSL translation teaching differ significantly, further investigation is imperative to assess the applicability and effectiveness of the continuation task approach in CSL contexts.

Based on the background outlined above, this study uses Khmer-Chinese translation as a case study to examine how the translation continuation task facilitates language learning and improves translation quality. The aim is to broaden the scope of application of the translation continuation task and to offer a fresh perspective for improving CSL translation teaching.

## 2. Literature Review

### 2.1. Continuation Argument

Although interaction and alignment in second language (L2) learning have garnered considerable attention, it represents a relatively new development that traces the genesis of interaction (Sato, 2017). Building on the interactive alignment model, Chuming Wang, a prominent Chinese professor specializing in L2 research, has proposed the Continuation Argument (C. Wang, 2016a, 2017, 2021). The term “continuation” refers to a speaker’s picking up another person’s turn during social interaction and creatively completing or expanding it, thereby responding to what has been said and setting up the next turn (C. Wang, 2016a, 2018). The Continuation Argument has two key premises. First, language is acquired through continuation (C. Wang, 2016a). Continuation typically takes place within interpersonal dialogues, where individuals can express their own views through understanding others’ speech. Continuation drives the interaction, and without it, there would be no interaction. Moreover, alignment is actually a learning effect (Atkinson et al., 2007). Second, high efficiency in language learning is achieved by continuation (C. Wang, 2016a). An inherent asymmetry exists between language comprehension and production, which serves as an incentive for triggering alignment (C. Wang, 2021). Continuation facilitates the intersection of language comprehension and production, gradually reaching stable language proficiency and achieving alignment through interaction. Therefore, the Continuation Argument can be viewed as a theoretical framework for language acquisition that emphasizes deepening interaction and alignment.

The Continuation Argument proposes new ideas and methods for enhancing language acquisition efficiency, demonstrating clear positive effects on learning. Its theoretical rationale is that continuation provides learners with linguistic templates for imitation and bases for content creation (C. Wang, 2021). (i) The continuation capitalizes on the asymmetry between language comprehension and language production. This asymmetry between language input and output naturally exists in human cognition and does not require artificial creation (C. Wang, 2021). Continuation tightly binds language input and output, triggering their interaction and flattening the language proficiency from lower to higher levels, thereby achieving the alignment. (ii) The continuation fully leverages contextual information. Continuation tasks require sufficient language input with rich contextual information, including linguistic expressions and situational cues. The creation of continuation texts builds upon established contexts from prior materials, enabling learners to easily activate and use previously acquired knowledge, thereby facilitating natural language acquisition (C. Wang, 2015). (iii) The continuation maximizes the alignment effect arising from interaction. During interaction, bidirectional alignment occurs between individuals, and unidirectional alignment occurs between individuals and materials (C. Wang & Wang, 2015). Without interaction, no alignment can be achieved (Pickering & Garrod, 2004), and the intensity of interaction is directly correlated with the degree of the alignment.

In short, language comprehension and production are two crucial parameters of the Continuation Argument, and their asymmetry is a prerequisite for its effectiveness. The continuation task serves as an intermediary between comprehension and production (C. Wang, 2023). Effective continuation-oriented tasks typically stimulate interaction between these two parameters, gradually fostering alignment and bridging the language gaps. This sheds new light on L2 translation teaching and learning, indicating an efficient approach for enhancing translation acquisition efficiency.

### 2.2. Translation Continuation Task

The Continuation Argument is primarily used in language acquisition research and focuses on designing various continuation-based tasks for language learning. Based on output modalities, continuation-oriented tasks can be classified into written continuation, spoken continuation and translation continuation (C. Wang, 2016a). Previous studies have primarily concentrated on the alignment effects in written and spoken continuation tasks, with the emergence of numerous achievements (e.g., C. Wang & Wang, 2015; Zeng et al., 2017; Li et al., 2020; Hong & Shen, 2021; H. Zhang, 2022; S. Zhang & Wang, 2023; Zhou & Wang, 2024). However, there is a lack of empirical evidence on the potential of the translation continuation task to facilitate language learning, warranting further exploration. What, then, is the translation continuation task? Its core mechanism is interaction and alignment, which refers to incorporating the element of continuation into language comprehension and production in translation (C. Wang, 2018). Generally speaking, the elements of “continuation” are primarily reflected in three aspects. First, in terms of content, the continuation should closely follow the plot thread of the original text (e.g., character actions, event developments, etc.), and expand upon, rather than merely repeat, the existing content. Second, in terms of language, it should emulate the stylistic features of the original text, including vocabulary choice and grammatical patterns, to maintain a consistent tone and register. Third, in terms of logic, it should follow the cause-and-effect relationships established in the original text and develop new plot elements naturally around the theme. The specific operational steps of the translation continuation task are outlined as follows: (i) Suitable bilingual reading materials, approximately 1000 words in length, are selected. The initial two-thirds serve as pre-reading material, while the remaining one-third is used for translation continuation. (ii) Learners are first provided with pre-reading materials and asked to produce their own translations. Then, they complete a bilingual reading activity to better understand the content. (iii) Learners complete the translation continuation task and compare their own translations with the corresponding published translations.

Currently, the translation continuation task and its pedagogical effects have attracted growing scholarly attention, accompanied by a steady increase in related achievements. Xu (2016) was the first scholar to conduct empirical research on the translation continuation task, indicating its significant alignment effect and improvement in translation quality. This study expanded the application scope of the continuation task, further supporting its efficacy. M. Wang and Ding (2022) and Yang and Hu (2022) primarily examined the alignment effect of the translation continuation task in professional English translation teaching. The former emphasized that multiple rounds of translation continuation facilitated learners’ internalization of translation knowledge and improvement of their translation skills, while the latter reported that interaction and alignment effects were mainly observed at the phrase level and showed an increase in the learners’ translation skills. Yuan and Chen (2024) viewed the translation continuation task as a pedagogical approach and explained its instructional mechanism and promotion effects through activities such as reading, continuation and translation, thereby highlighting a clear alignment effect. Wu et al. (2025) examined the task motivation of EFL learners in the translation continuation task based on the Continuation Argument and found that topic selection and content features were the most influential factors.

Obviously, some research has been conducted on the translation continuation task, and its impact on facilitating ESL learning is constantly being examined, holding implications for practices in translation teaching. Nevertheless, little attention has been given to exploring its effectiveness in the CSL field, resulting in scant studies on how the translation continuation task promotes CSL translation learning and teaching. As challenges in CSL translation teaching persist, it becomes critical to introduce new approaches that address these issues while enhancing overall teaching and learning efficiency. Therefore, this warrants further investigation into this research interface and “black box”.

## 3. Methodology

A case-based quantitative research study was designed, supplemented by qualitative analysis. The data were primarily collected through empirical research in a CSL translation class, with SPSS 28.0 being used for data processing. Qualitative analysis was utilized to discuss the attitudes of both teachers and students towards the translation continuation task. The first-hand data enables us to gain a deep understanding of the linguistic features observable in the translation continuation task and their potential pedagogical implications.

### 3.1. Research Aim and Questions

In light of the Continuation Argument, a theoretical model positing that interaction and alignment facilitate language acquisition, this study investigates the linguistic phenomena observed in the translation continuation task. Rather than presupposing interactive alignment as the only mechanism, this study treats the observable “alignment phenomenon”, namely the reuse of linguistic forms from pre-reading materials, as a descriptive starting point for the investigation. The primary aim is to systematically describe the features of this phenomenon and to explore its correlation with translation output quality. Specifically, the following three questions are addressed.

(1) To what extent do learners reuse target words, grammatical structures, and discourse cohesive devices from the pre-reading materials during the translation continuation task?
(2) Is there a correlation between the translation continuation task and the quality of the CSL translation output?
(3) How do teachers and students perceive the use of the translation continuation task?

### 3.2. Participants

This study was conducted at a Chinese school in Phnom Penh, Cambodia. The school comprises an early childhood department, a primary department (Grades 1 to 6) and a secondary department (Grades 7 to 9), which implements half-day teaching with Chinese immersion. The courses offered include Chinese language, Cambodian-Chinese translation, mathematics, history, and natural science. At present, there are over 1000 enrolled students and 35 teachers.

The participants are 60 CSL learners from two parallel Grade 8 classes, divided into groups A and B, with each group comprising 30 students. Participants in group A are numbered from 1 to 30, while participants in group B are numbered from 31 to 60. All participants are native Cambodian speakers aged between 15 and 18, including 41 female and 19 male learners. They have been learning Chinese for 6 years without any experience of studying in China. In other words, their educational background in Chinese is essentially similar. Additionally, the participants have been engaging in weekly sessions of Khmer-Chinese translation for two years (45 min per session). Before starting this study, the participants had acquired about 2300 Chinese words and possessed a Chinese proficiency of HSK Level 5. In the latest Chinese test, Group A (M = 89.08, SD = 5.92) and Group B (M = 88.72, SD = 5.07) obtained similar scores. An independent-samples *t*-test indicated no statistically significant difference in scores between the groups (t = 0.23, df = 58, *p* > 0.05, 95% CI [−2.51, 3.18]). The 95% confidence interval for the mean difference ranged from −2.51 to 3.18 points and the small effect size (Cohen’s d < 0.2) further suggests that any true difference is likely to be small. Therefore, all participants reached a similar Chinese proficiency at the time of testing.

### 3.3. Materials

The operational procedures involved in the translation continuation task were comprehensively illustrated by C. Wang (2018). By integrating Wang’s steps with Krashen’s (1985) “i + 1” principle, this study selects two Khmer-Chinese translated articles from extracurricular reading materials (Middle school edition) as experimental materials, both of which are narrative essays. The first one is “Father’s Love” (父爱), showcasing the profound affection a father has for his son. It contains 7 paragraphs and a total of 1497 characters. The second one is “Old Charcoal Seller” (卖炭翁), depicting the harsh lives of lower-class workers in ancient China. It comprises 8 paragraphs and a total of 1144 characters. More than 80% of the vocabulary used in both texts has been acquired beforehand. The language difficulty (LD) is assessed by Chinese Text Compass, and the result indicates that LD ranges from 3.00 to 3.50, which is equivalent to HSK Level 5 and aligns with the participants’ Chinese proficiency. Based on this, target investigation content includes 15 words, 4 grammar points and 4 types of discourse cohesive devices.

### 3.4. Procedures

**Figure 1:**
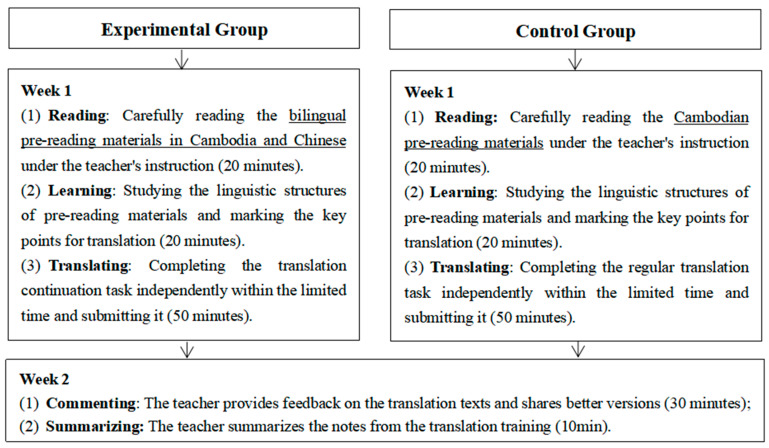
The design of the empirical process.

The entire empirical study consists of two rounds, each lasting for two weeks. Each participant is required to complete two tasks: one for the continuation translation task and the other for the regular translation task. In the first round (week 1 to 2), group A serves as the experimental group and completes a translation continuation task, while group B serves as the control group and completes a regular translation. The experimental text used is “Father’s Love”. The initial five paragraphs are bilingual pre-reading materials in Chinese and Khmer, containing 789 Khmer words (1006 Chinese characters), accounting for 67.70% of the whole text. The last two paragraphs are the content to be translated, totaling 95 Khmer words. In the second round (week 3 to 4), group B becomes the experimental group and completes the translation continuation task, while group A is the control group and finishes a regular translation task. The experimental text is “Old Charcoal Seller”. Similarly, the first five paragraphs serve as pre-reading materials and include 556 Khmer words (739 Chinese characters), accounting for 65.00% of the full text. Furthermore, there are two final paragraphs that serve as materials requiring translating, with a total of 296 Khmer words. The specific process is shown in Figure 1.

There are three points that require clarification. First, only the experimental group received bilingual pre-reading materials, whereas all other conditions were identical between the two groups. Second, the same teacher conducted the experiment, thereby minimizing variations in instructional delivery. Third, during the class, the teacher did not inform participants in advance which language points would be the focus; instead, the teacher merely guided learners to read the pre-reading materials, clarified their meanings, and reminded them to pay attention to the language forms therein, without permitting copying or plagiarism.

After completing this empirical study, we examined the participants’ and the teachers’ attitudes towards the translation continuation task through questionnaires and interviews.

### 3.5. Data Collection and Processing

A total of 120 translation texts were collected in this study, comprising 60 continuation translation texts and 60 regular translation texts. After repeated inspection, all the texts were found to be free of plagiarism and are therefore deemed valid. The text processing was conducted in three steps to operationalize and measure the alignment phenomenon. Firstly, the 120 texts were converted into electronic form one by one and coded according to group numbers, experimental rounds and participant numbers, establishing a mini-corpus. Secondly, the Chinese Research Helper was used for word segmentation, sentence segmentation and part-of-speech tagging. Then, the results were manually proofread. Thirdly, and crucially for addressing research question 1, a careful comparison was conducted between the pre-reading translated materials and learners’ output texts. The usage frequency of target words, grammatical points, and discourse cohesive devices was counted. This quantitative measure serves as our operational indicator of the “alignment phenomenon” observed in the translation continuation task. It is important to note that this measure captures surface-level similarity, which may arise from multiple mechanisms, including, but not limited to, priming or strategic borrowing.

### 3.6. Scoring Criteria

Due to the absence of systematic and operational scoring criteria for Khmer-Chinese translation, this study adopted the grading standards used in the English-Chinese translation section of China’s College English Test. The specific details (see Table 1) were collaboratively determined by the classroom teacher together with two other experienced teachers proficient in Khmer-Chinese translation.

The other two teachers involved in preparing the scoring criteria were invited to score the translation texts. Generally, each text’s final score was determined by calculating the arithmetic mean of two scores assigned by these teachers. If the difference between the two scores exceeded three points, a third teacher was invited to rescore the text, and the final score was then calculated as the arithmetic mean of all three scores.

## 4. Results and Analysis

### 4.1. Statistics on Translation Text Scores

The SPSS 28.0 software was used to perform Pearson correlation and reliability tests on the scores given by different teachers. The findings revealed a significant positive correlation, high reliability and strong internal consistency of scores (r = 0.85, *p* < 0.001; Cronbach’s Alpha = 0.89). Table 2 presents the translation scores for participants in both the experimental and control groups.

According to Table 2, it can be seen that there are score differences between the two groups. The experimental group demonstrates higher scores compared to the control group, indicating that translation continuation texts yield superior scores to regular translation texts. The results of an independent-samples *t*-test revealed statistically significant differences in scores between these two groups in both rounds (Round 1: t = 10.88, df = 58, *p* < 0.05, 95% CI [5.48, 7.95], Cohen’s d > 1; Round 2: t = −12.67, df = 45.42, *p* < 0.05, 95% CI [−8.70, −6.33], |Cohen’s d| > 1), with the experimental group consistently outperforming the control group.

Specifically, there are some low-frequency words in the two pre-reading materials, including *gudie* (姑爹, uncle-in law), *laoweng* (老翁, old man), *yaochuan* (摇船, row a boat), *jishi* (集市, bazaar) and youhei (黝黑, swarthy), which are relatively difficult to understand and translate accurately. Especially for participants in the control group who lack Chinese pre-reading materials, a higher error rate is observed in translating these words. For instance, in the control group, 21 participants mistakenly translated *gudie* (姑爹) as *shushu* (叔叔, uncle) or *jiujiu* (舅舅, uncle), while 20 participants inaccurately translated *shougulinxun* (瘦骨嶙峋, scraggy) as miaotiao (苗条, slim). Furthermore, the frequency of using cohesion modes by participants in the control group was relatively low. For example, the mode of ending being correlated with the beginning was only utilized three times. Therefore, the scores of translation texts within the control group were relatively lower, indicating poor quality. In contrast, Khmer and Chinese pre-reading materials were provided to the experimental group, enabling participants to repeatedly read bilingual texts and become familiar with target words, grammar points and discourse cohesive devices. Hence, the frequency of using target language points was higher in the experimental group, resulting in higher scores. It can be inferred that the translation continuation task effectively improves both translation text scores and Chinese translation proficiency.

### 4.2. Alignment in Different Language Levels

Table 3 presents the reuse frequency of target words, grammatical structures, and cohesive devices in translation continuation tasks across two groups.

To compare performance on translation tasks between the experimental and control groups, a series of independent-samples *t*-tests were conducted using SPSS 28.0. The analyses focused on three linguistic dimensions: target word reuse, grammatical point reuse, and discourse cohesion device reuse. (1) Overall group difference. Analysis of combined reuse frequency from both rounds revealed that the experimental group exhibited significantly higher reuse frequency than the control group across all dimensions (words: t = 2.98, df = 28, *p* = 0.006, 95% CI [5.69, 30.71], Cohen’s d > 1; grammatical points: t = 4.09, df = 6, *p* = 0.006, 95% CI [3.62, 14.38], Cohen’s d > 1; discourse cohesion: t = 2.48, df = 6, *p* = 0.048, 95% CI [0.29, 49.71], Cohen’s d > 1). Notably, the lower degrees of freedom for the grammar and discourse analyses (df = 6) resulted from the Welch-Satterthwaite approximation, which was applied because Levene’s test indicated unequal variances (*p* < 0.05). (2) Differences in Each Translation Round. In the first round, the experimental group significantly outperformed the control group on all measures (words: t = 11.47, df = 44.58, *p* = 0.000, 95% CI [3.30, 30.71], Cohen’s d > 1; grammatical points: t = 4.59, df = 44.70, *p* = 0.000, 95% CI [0.37, 0.96], Cohen’s d > 1; discourse cohesion: t = 9.20, df = 58, *p* = 0.000, 95% CI [1.83, 2.84], Cohen’s d > 1). In the second round, the reuse frequency of the experimental group was significantly higher than that in the control group (words: t = −9.33, df = 49.51, *p* = 0.000, 95% CI [−6.19, −4.00], |Cohen’s d| > 1; grammatical points: t = −2.61, df = 58, *p* = 0.012, 95% CI [−0.94, −0.12], |Cohen’s d| > 0.6; discourse cohesion: t = −6.16, df = 58, *p* = 0.000, 95% CI [−1.33, −0.67], |Cohen’s d| > 1). Therefore, it can be seen that the translation continuation task facilitates the reuse of target language structures from the source texts, thereby triggering interactive alignment in language use.

Additionally, this study conducted a detailed analysis of the usage of target words, grammatical points and discourse cohesive devices in two groups (see Table 4) for further discussion.

Firstly, it is evident from Table 4 that the frequency of the target words used by the experimental group exceeds that of the control group. For instance, there is a substantial disparity in reuse frequency of words such as *laoweng* (老翁), *gudie* (姑爹) and *jishi* (集市). The most striking evidence of lexical alignment is observed with the word *laoweng* (老翁), where the experimental group used it significantly more often than the control group (68 vs. 11). This difference is not merely quantitative but also a qualitative indicator of successful uptake. The word *laoweng* (老翁) embodies stylistic features of classical Chinese writing that are relatively uncommon in modern Chinese. If there is no advance exposure, accurate translation becomes challenging for participants in the control group. The translation continuation task operationalized input enhancement and frequency effects by embedding this word 13 times in the pre-translated text, thereby directing learners’ focal attention to this otherwise low-salience word. Consequently, learners did not merely notice it; they incorporated it into their own translation output, demonstrating a direct form of lexical priming and alignment with the source text. Another example illustrating this phenomenon is observed with the word *kaoqu* (考取), which appeared 11 times in the experimental group compared to only once in the control group. This clearly indicates the translation continuation task facilitates learning.

Secondly, the differences in the reuse of target grammatical structures reveal a process of syntactic priming and selective alignment induced by the continuation task. Taking the degree complement as an example, the experimental group reused it 13 times (all correct), significantly more than the control group (2 instances). This divergence underscores a key mechanism of the task: by directly linking comprehension to production, it enhances the salience of input forms and increases the probability of their reactivation in subsequent output. Thus, the higher frequency and accuracy reflect incidental acquisition through alignment with the input, demonstrating that the task effectively guides learners’ grammatical choices toward those of the input model.

Thirdly, discourse-level analysis reveals that the translation continuation task effectively scaffolds the acquisition and deployment of cohesive devices, thereby enhancing textual coherence. This study focused on four discourse cohesive devices in its investigations. The experimental group showed a significantly higher use of all four targeted cohesive devices, most notably omission (57 vs. 14 instances) and the rhetorically more complex ending-correlated-with-beginning device (9 vs. 3 instances), suggesting that the task does more than merely raise awareness. By providing a coherent textual model, the task offers learners a template for achieving cohesion. For simpler devices such as omission, this likely facilitates direct procedural imitation. For complex devices, it may support a deeper understanding of rhetorical function, enabling learners to adapt the underlying principle to their own output.

In summary, the bilingual materials in Khmer and Chinese provide participants in the experimental group with a language imitation template and a content creation basis during translation, facilitating a close combination of language input with output. This is indicated by utilizing more target words, grammar and discourse modes from pre-reading materials to support subsequent translation. Consequently, the output language aligns with the input language, inducing an increase from the lower to the higher language level (C. Wang, 2012). It can then be inferred that the translation continuation task effectively triggers interaction and alignment in language use.

### 4.3. Attitudes Towards the Translation Continuation Task

The survey results indicate that both teachers and students generally hold a positive attitude towards the translation continuation task. They believe that this approach effectively integrates language input and output, yielding benefits for language learning. The details are as follows.

Firstly, bilingual pre-materials facilitate the reuse of target language forms in translation continuation tasks. A majority of teachers and students (87.30%) believe that providing texts in both Khmer and Chinese during the pre-reading stage is helpful. The corresponding Chinese translations capture learners’ attention to language features, enabling them to imitate linguistic expressions and thereby lay a foundation for translation continuation tasks. For instance, participant No. 29 from group A stated, “When reading texts in both Khmer and Chinese, I pay more attention to word and sentence translations and imitate the expressions, facilitating rapid acquisition of the usage”. This remark links bilingual input to metalinguistic noticing and imitation—two key cognitive processes central to the task’s pedagogical effectiveness. Similarly, participant No. 15 in group B expressed, “Reading bilingual materials really helped me understand the content. Once understanding is achieved, translations become somewhat smoother”. This highlights that bilingual support facilitates language comprehension, thereby reducing cognitive load during translation and reinforcing the interconnection between understanding and production.

Secondly, the translation continuation task facilitates the improvement of translation skills. All three teachers in this study agree that it is an effective approach to enhancing translation learning. For instance, teacher No. 1 emphasized, “The experimental group demonstrated a higher quality of translated texts, exhibiting a noticeable increase the reuse of target words, grammatical points and discourse cohesive devices, which can mainly be attributed to the translation continuation task”. This observation links the task to measurable linguistic outcomes, supporting its role in promoting linguistic alignment. Moreover, 78.33% of participants also believe that the translation continuation task contributes to improving their translation level.

Thirdly, the implementation of the translation continuation task can be further enhanced in Chinese translation classrooms. The teachers fully acknowledge that the efficiency of this teaching method facilitates learning and advocate its sustainable use in Khmer-Chinese translation classrooms. For instance, teacher No. 2 mentioned, “The impact of the translation continuation task is evident, hence it deserves promotion”. This reflects observed classroom effectiveness, reinforcing the task’s perceived instructional value. Furthermore, over 60% of the participants expressed their desire to continue adopting this approach in translation learning. Participant No. 3 from group A commended the practice of first reading bilingual materials and then translating, hoping for its sustained usage by the teacher. Such feedback reflects learners’ acceptance of the translation continuation task, which can foster engagement and autonomous skill development.

In a word, the translation continuation task provides participants with language translation templates and the opportunities to closely imitate the language structures in pre-reading texts. This facilitates their comprehension of the content and improves their translation skills. Consequently, both teachers and participants hold a positive attitude toward the translation continuation task.

## 5. Discussion

Guided by the Continuation Argument, this paper focuses on the translation continuation task and conducts an empirical study using Khmer-Chinese translation as an example. The findings demonstrate that: (i) The translation continuation task effectively promotes interaction and alignment between CSL learners’ language use and the source text. (ii) The translation continuation task accelerates the initiation of language structures and improves the quality of translation texts. (iii) Both teachers and students hold a positive attitude towards the translation continuation task, recognizing its effectiveness in promoting learning. Combined with the experimental results, the discussions are as follows.

### 5.1. Interpretations of the Language Alignment

The key contribution of this study is to observe and quantify a measurable alignment phenomenon—namely, the increased reuse of linguistic forms from the source text in the translation continuation task. Although documenting this phenomenon is crucial, it is equally important to distinguish it from claims about its underlying cognitive mechanisms. Several interpretations are possible, but the design of this study does not allow us to identify a single cause.

First, the possibility of pedagogically induced imitation was reduced, as the teacher directed learners’ attention to language features without explicitly identifying the target language items. However, another plausible explanation for the reuse of language is short-term priming (Pickering & Garrod, 2004). The task design of translation continuation following the reading of bilingual materials ensured that recently processed words and grammatical structures were highly activated in memory, making them easily retrievable and usable. This priming effect was likely augmented by task-induced strategic borrowing, in which learners consciously drew upon the pre-reading text as a contextualized linguistic resource.

Based on the Continuation Argument, linguistic-level alignment can be viewed as a manifestation of a more profound interactive alignment process (C. Wang, 2018). However, it should be emphasized that the translation continuation task can, to a certain degree, provide evidence for the theoretically more comprehensive, meaning-negotiation-based mechanism of interactive alignment. Yet it is not the only evidence. The alignment observed in this study could also be accounted for by priming and strategic borrowing. The potential role of interactive alignment as a sustained, deeper learning mechanism facilitated by “continuation” remains a valuable hypothesis for future longitudinal research.

### 5.2. Potential Explanations for Task Effectiveness

The alignment phenomenon and correlated output quality observed in this study may be attributed to several design features of the translation continuation task.

Firstly, the translation continuation task is designed to create a coherent context that motivates focused engagement. The requirement to produce a translation continuation that is coherent with the provided narrative establishes a clear communicative goal (Tomasello, 2001), shifting the task from decontextualized translation to a goal-oriented, meaning-construction activity. This structural feature is theorized to enhance task-induced involvement (Laufer & Hulstijn, 2001), which encompasses both cognitive (e.g., attention to linguistic form) and affective (e.g., motivation to complete the story) dimensions. In this study, participants in the experimental group received bilingual (Khmer and Chinese) pre-reading materials. To produce a coherent continuation, they needed to process these materials not only for comprehension but also as linguistic and stylistic resources for their own translation output. This likely prompted deeper processing and selective attention to the language forms in the source texts—forms that were relevant to the immediate production goal. As a result, learners reused target words, grammatical structures, and cohesive devices from the source texts, thereby enhancing linguistic alignment. This observed linguistic alignment (i.e., increased target language form reuse) can be interpreted from the perspective of the Continuation Argument as potentially reflecting a deeper process of interactive alignment driven by task-induced engagement. However, as noted in Section 5.1, alternative explanations, such as priming and strategic borrowing, remain equally plausible based on the current data.

Secondly, the translation continuation task integrates language comprehension and production. “Continuation” closely bridges the input and output (C. Wang, 2012). In the experimental group, participants read parallel Khmer-Chinese texts before completing the translation continuation task, thereby reinforcing their understanding of Chinese words and grammatical structures. During continuation translation, participants interacted with known Chinese texts and learned local language expressions, such as word collocations, sentence structures, and discourse devices, then applied them in their own translations. This promoted alignment of the output language with the input language, with a gradual flattening effect from lower to higher levels (C. Wang, 2012). Therefore, the experimental group used target words, grammar points and discourse cohesive devices more frequently, and their translation texts were closer to the original text. Here are two examples for further explanation.
Example I (Experimental group)为了节省钱，爸爸再向姑爹借渔船，两人摇船送我到学校。Wei le jiesheng qian, baba zai xiang gudie jie yuchuan, liang ren yaochuan song wo dao xuexiao.In order to save money, my father borrowed a fishing boat from my uncle-in law, and they rowed me to the school by the boat.Example II (Control group)为了不花钱，爸爸向叔叔借了船，他们用船送我去上学。Wei le bu hua qian, baba xiang shushu jie le chuan, tamen yong chuan song wo qu shangxue.In order to save money, my dad borrowed a boat from my uncle, and they sent me to school by boat.

In Example I, there are four target words and one discourse cohesive device (substitution), all of which are used correctly. Conversely, in Example II, apart from the substitution mode, none of the target words is used accurately. These two examples present a stark contrast, and Example I is more precise and stylistically consistent with the source text.

Thirdly, the translation continuation task promotes noticing. Ellis (2005) noted that noticing helps learners consciously associate language forms with textual features. The continuation task bridges input and output, directing attention to learners’ awareness of gaps between interlanguage and the target-like models in the text (Swain, 1985), highlighting the contrast effect. The experimental group employed the approach of the translation continuation task and were provided bilingual pre-reading materials. This helps learners internalize standardized Chinese expressions, recognize gaps between their proficiency and classical translations, and build awareness of those gaps. Through extensive reading of high-quality Chinese translations, participants gradually developed a model of excellent works in their minds (C. Wang, 2018). During continuation translation tasks, participants imitated the Chinese source texts and applied translation skills to overcome expression barriers, aligning their translated output with the original materials, thereby bridging the language gaps and addressing textual deficiencies (C. Wang, 2023). The results show the experimental group outperformed the control group both qualitatively and quantitatively in target word use, grammar accuracy, and discourse coherence.

From the above, it can be seen that the design features of the translation continuation task facilitate the observed alignment phenomenon across linguistic levels. In this study, this phenomenon is most directly manifested in the increased reuse of target words, grammatical structures, and discourse cohesive devices from the source text, which can be viewed as a form of textual adaptation. In contrast, while completing the task, learners also use new language forms and refine the content of their translations, which suggests a degree of creative application. Whether these surface-level manifestations reflect the deeper, sustained cognitive processes theorized under constructs such as adaptive or creative alignment requires further investigation.

### 5.3. Contextual Scaffolding and Output Quality

Furthermore, the translation continuation task offers rich contextual information, aligning with the principle of Learn-Together-Use-Together. Learn-Together refers to language learning through interaction and understanding, while Use-Together pertains to language production and acquisition (C. Wang, 2011). Acquiring a language within an appropriate context facilitates the initiation and accurate application of learned language structures (C. Wang, 2016b). The translation continuation task provides abundant and effective contextual support for learning Chinese language structures, which can serve as a situational scaffold (Yuan & Chen, 2024), making the meaning and usage of linguistic structures more transparent. This implies that understanding both the content and context of the original text becomes more effective. Simultaneously, thanks to the correct Chinese context accompanying the translation continuation task, contextual markers have been attached to the target knowledge. This can be understood from two perspectives. On the one hand, it encourages participants to actively utilize language forms in the original texts, enhancing vocabulary acquisition as well as grammatical and discourse expression abilities (C. Wang, 2016a). On the other hand, the provided correct language templates enable participants to borrow accurately within a meaningful framework, effectively reducing the likelihood of errors (Q. Wang & Wang, 2016; H. Zhang, 2022). This directly contributes to the higher translation quality scores in the experimental group. The positive attitudes reported by teachers and students are consistent with this view of the task as a supportive and effective pedagogical tool.

In conclusion, this study observed and quantified a consistent alignment phenomenon: the increased reuse of linguistic forms from the source text in the translation continuation task. In this task, the “continuation” functions as a bridge that integrates textual engagement with content creation. Supported by extensive contextual input, it facilitates interactive alignment between learners’ output and input, which is associated with higher translation quality in this study. These findings position the translation continuation task as a promising pedagogical context for inducing measurable language alignment, the deeper cognitive nature of which (e.g., whether it constitutes sustained interactive alignment) remains a vital question for future research.

## 6. Conclusions

This study establishes an observable and measurable framework for investigating language alignment in the translation continuation task. The findings demonstrate that this task has a positive impact on CSL translation learning and output quality, as evidenced by increased linguistic alignment and higher scores. Both teachers and learners recognize its potential as a supportive pedagogical tool. Based on these findings, this paper proposes several practical suggestions for CSL translation teaching.

First and foremost, the principle of “close combination between language comprehension and production” should be consistently applied in CSL translation teaching. Within the premise of “i + 1”, teachers should strive to provide learners with sufficient translation templates while appropriately increasing language input. Meanwhile, learners are also encouraged to practice more translation to enhance Chinese language proficiency.

Secondly, the CSL translation classroom should actively promote the translation continuation task. In class, the teacher provides learners with bilingual pre-reading materials and guides them to study effectively. This approach facilitates the acquisition of language knowledge and the construction of situational patterns consistent with the original texts, promoting multiple interactions and alignments, thereby showing potential for improving both translation quality and teaching effect. It is important to reiterate that the current study primarily provides a descriptive account of the alignment phenomenon in the task context.

In addition, there are certain limitations to this study. The first one is the confounding of variables in the design: the experimental condition differed from the control in two respects, which prevents isolating the only effect of either factor. Future research will include the necessary third condition (bilingual input with regular translation) to disentangle these effects and rigorously test the causal claim. The second limitation is that this study did not examine the transferability of the observed learning advantage. While it quantified the immediate facilitative effect within the task, it remains unclear whether this benefit transfers to new contexts (e.g., inferring meanings of unseen vocabulary) or persists over time. Future research will incorporate transfer tasks to assess learners’ generalized strategic competence. The third limitation is that this study did not examine the restructuring of learners’ interlanguage due to the short investigation duration. Future studies could extend the investigation period and adopt multiple control groups to examine how interlanguage evolves across successive translation continuation tasks. The final limitation is that this study did not investigate the underlying cognitive mechanisms (such as alignment), which will be explored in future research using methods such as think-aloud protocols.

## Figures and Tables

**Table 1:** Scoring rules.

Grade	Score (S)	Detailed Rules
A	72 ≤ S ≤ 80	Accurately translating the text, maintaining a consistent language style and tone, smoothly using target words, grammatical points and discourse cohesive devices, with only a few minor errors.
B	64 ≤ S < 72	Essentially translating the text, being coherent and fluid in writing, without major language errors.
C	56 ≤ S < 64	Translating half of the text, using inappropriate words and making numerous language mistakes.
D	48 ≤ S < 56	Translating less than half of the original text, using inaccurate words, with quite a few serious language mistakes.
E	32 ≤ S < 48	Only translating some words and sentences accurately, with fragmented translations, barely expressing the original text meaning.
F	S < 32	With only a few words translated or an unrelated translation text.

Note: Score out of 80.

**Table 2:** Scores of translation texts.

Rounds	Experimental Group				Control Group			
	Mean	Max	Min	Std.	Mean	Max	Min	Std.
1	68.68	72.00	64.25	1.83	61.96	69.25	55.50	2.84
2	70.35	73.00	66.25	1.57	62.83	68.75	57.25	2.85

**Table 3:** The reuse of language forms.

		Round 1		Round 2	
		Mean	Std.	Mean	Std.
Words	Experimental Group	5.73	1.68	9.93	2.52
	Control group	1.73	0.91	4.83	1.62
Grammatical points	Experimental Group	0.83	0.70	0.97	0.93
	Control group	0.17	0.38	0.43	0.63
Discourse cohesive devices	Experimental Group	3.10	1.18	1.47	0.68
	Control group	0.77	0.73	0.47	0.57

**Table 4:** Statistics on the usage of target language points in two groups.

Language Levels	Target Points	Experimental Group Frequency (Number of Participants)	Control Group Frequency (Number of Participants)	Frequency Difference (Number of Participants)
Words	weiyi (只有, only)	24 (21)	7 (7)	14 (14)
	yuchuan (渔船, fishing boat)	16 (16)	3 (3)	13 (13)
	yaochuan (摇船, row a boat)	19 (17)	4 (4)	15 (13)
	jiesheng (节省, save)	27 (23)	11 (11)	16 (12)
	kaoqu (考取, pass)	11 (11)	1 (1)	10 (10)
	suiran (虽然, though)	28 (23)	17 (17)	11 (6)
	gudie (姑爹, uncle-in-law)	47 (30)	9 (9)	38 (21)
	jishi (集市, bazaar)	37 (29)	17 (17)	20 (12)
	laoweng (老翁, old man)	68 (30)	11 (11)	57 (19)
	banbai (斑白, grizzled)	19 (19)	7 (7)	12 (12)
	kesou (咳嗽, cough)	59 (30)	49 (30)	10 (0)
	tan (炭, carbon)	62 (30)	44 (30)	18 (0)
	youhei (黝黑, swarthy)	17 (17)	6 (6)	11 (11)
	shougu linxun (瘦骨嶙峋, scraggy)	15 (14)	3 (3)	12 (11)
	danbo (单薄, thin)	21 (18)	8 (8)	13 (10)
Grammar points	Bi-constituent construction	12 (12)	3 (3)	9 (9)
	Degree complement	13 (13)	2 (2)	11 (11)
	Bei (被)—Sentence	11 (11)	4 (4)	7 (7)
	V + le (了) V structure	18 (16)	9 (9)	9 (7)
Discourse Cohesion modes	Omission	57 (30)	14 (14)	43 (16)
	Conjunction	36 (30)	9 (9)	25 (21)
	Substitution	35 (26)	11 (11)	24 (15)
	the ending being correlated with the beginning	9 (9)	3 (3)	6 (6)

## Data Availability

The data presented in this study are available on request from the corresponding author due to privacy reasons.
